# Experimental tests of bivalve shell shape reveal potential tradeoffs between mechanical and behavioral defenses

**DOI:** 10.1038/s41598-020-76358-x

**Published:** 2020-11-10

**Authors:** Erynn H. Johnson

**Affiliations:** 1 Department of Earth and Environmental Science, University of Pennsylvania, 240 S. 33rd St., Philadelphia, PA 19104 USA; 2 Paleontological Research Institution, 1259 Trumansburg Road, Ithaca, NY 14850 USA

**Keywords:** Evolutionary ecology, Palaeoecology, Experimental evolution, Palaeontology

## Abstract

Bivalves protect themselves from predators using both mechanical and behavioral defenses. While their shells serve as mechanical armor, bivalve shells also enable evasive behaviors such as swimming and burrowing. Therefore, bivalve shell shape is a critical determinant of how successfully an organism can defend against attack. Shape is believed to be related to shell strength with bivalve shell shapes converging on a select few morphologies that correlate with life mode and motility. In this study, mathematical modeling and 3D printing were used to analyze the protective function of different shell shapes against vertebrate shell-crushing predators. Considering what life modes different shapes permit and analyzing the strength of these shapes in compression provides insight to evolutionary and ecological tradeoffs with respect to mechanical and behavioral defenses. These empirical tests are the first of their kind to isolate the influence of bivalve shell shape on strength and quantitatively demonstrate that shell strength is derived from multiple shape parameters. The findings of this theoretical study are consistent with examples of shell shapes that allow escape behaviors being mechanically weaker than those which do not. Additionally, shell elongation from the umbo, a metric often overlooked, is shown to have significant effects on shell strength.

## Introduction

Shells armor bivalves against both vertebrate predators (e.g., shell-crushing fishes, birds, and mammals) and invertebrate predators (e.g., drilling gastropods and cephalopods, asteroids and gastropods which attack at the shell margin, and shell-crushing arthropods), providing structural protection and permitting escape and/or avoidance behaviors^1–6^. The shapes of bivalve shells are often indicative of life mode: whether the organism lives above or below substrate, how it feeds, and how or if it locomotes^7,8^. The relatively simple but highly diverse shapes of bivalve shells are frequently used to illustrate the concept of functional morphology—the idea that morphology can be attributed to ecological role^9–13^. In fact, the ecological and evolutionary constraints on bivalve shell shapes are so restrictive that even very distantly related bivalves living in similar environments show convergence on a limited range of morphologies^7,8,14,15^. For example, burrowing bivalves tend to have thinner shells than bivalves that sit atop sediment^7,8^. While burrowing bivalves come in many shapes, shell elongation, resulting in reduced contact area with sediment during burrowing, is very common. Elongation is also frequently seen in taxa which attach to rocks or substrate, as an increased ventral portion of the shell provides area for stable attachment^8^. Swimming bivalves, which regularly rest on the sediment (e.g., pectinids), have thin, compressed shells that permit lift^16^. While thin shells are necessary for locomotion, both above and below the sediment, it is well understood that thinner shells are weaker and more easily penetrated by predators including shell-crushers^17–22^. Contrarily, bivalves that are highly vulnerable to predation (lacking means of hiding by burrowing or nestling) tend to have thicker, heavier shells^23^. Therefore, a clear tradeoff exists when creating a thin shell that is well adapted for escape or evasion of predators but provides less protection during an attack. Similar tradeoffs likely exist between bivalve shell geometries that permit different defensive strategies: namely those that serve primarily as mechanical armor and those which enable behavioral defenses.

**Figure 1:**
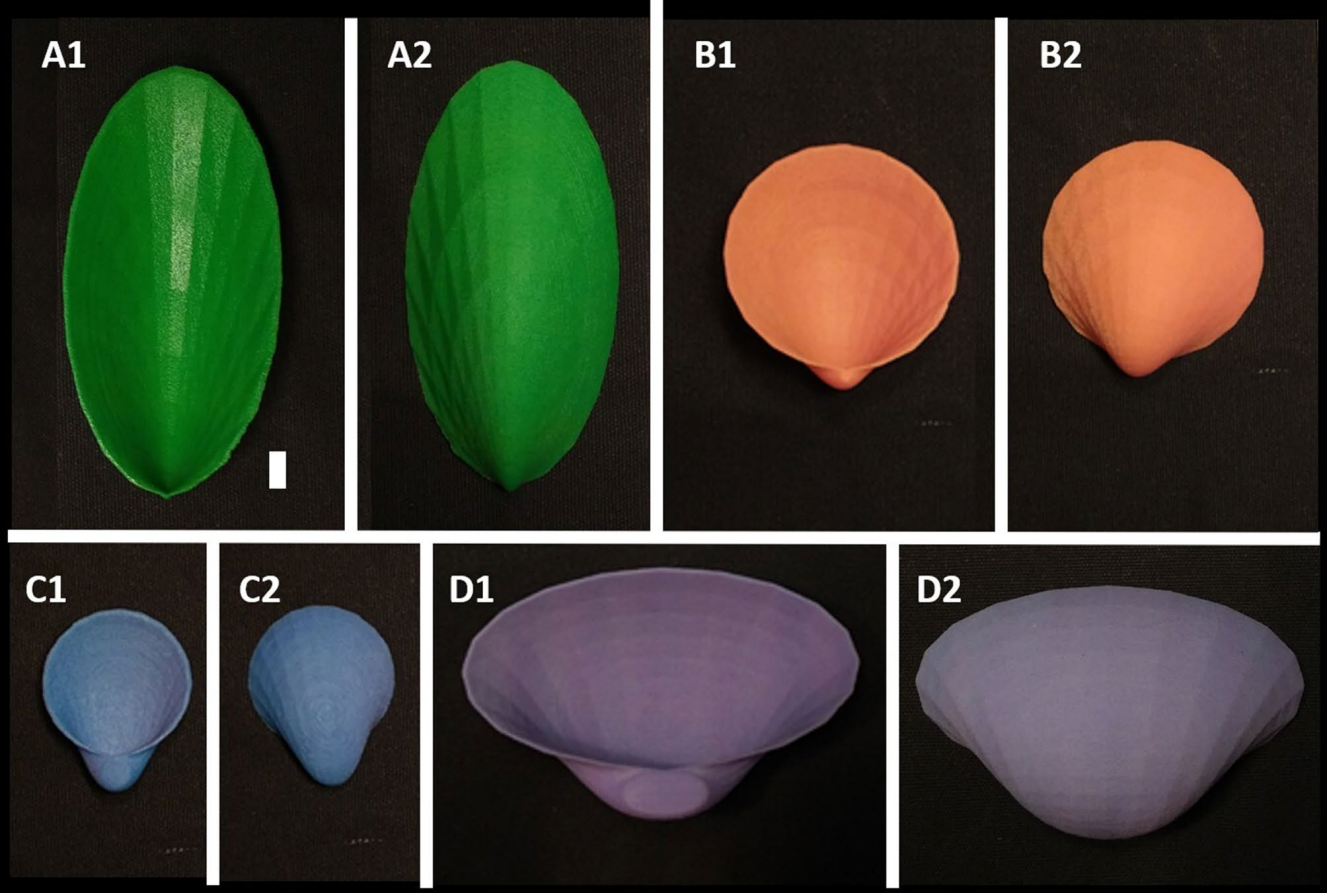
3D printed bivalve shell models: (**A**) G_2_W_2_; (**B**) 20G_1_W_1_, (**C**) 10G_1_W_0.5_, (**D**) G_0.5_W_0.5_ scale bar is 1 cm for all images.

**Figure 2:**
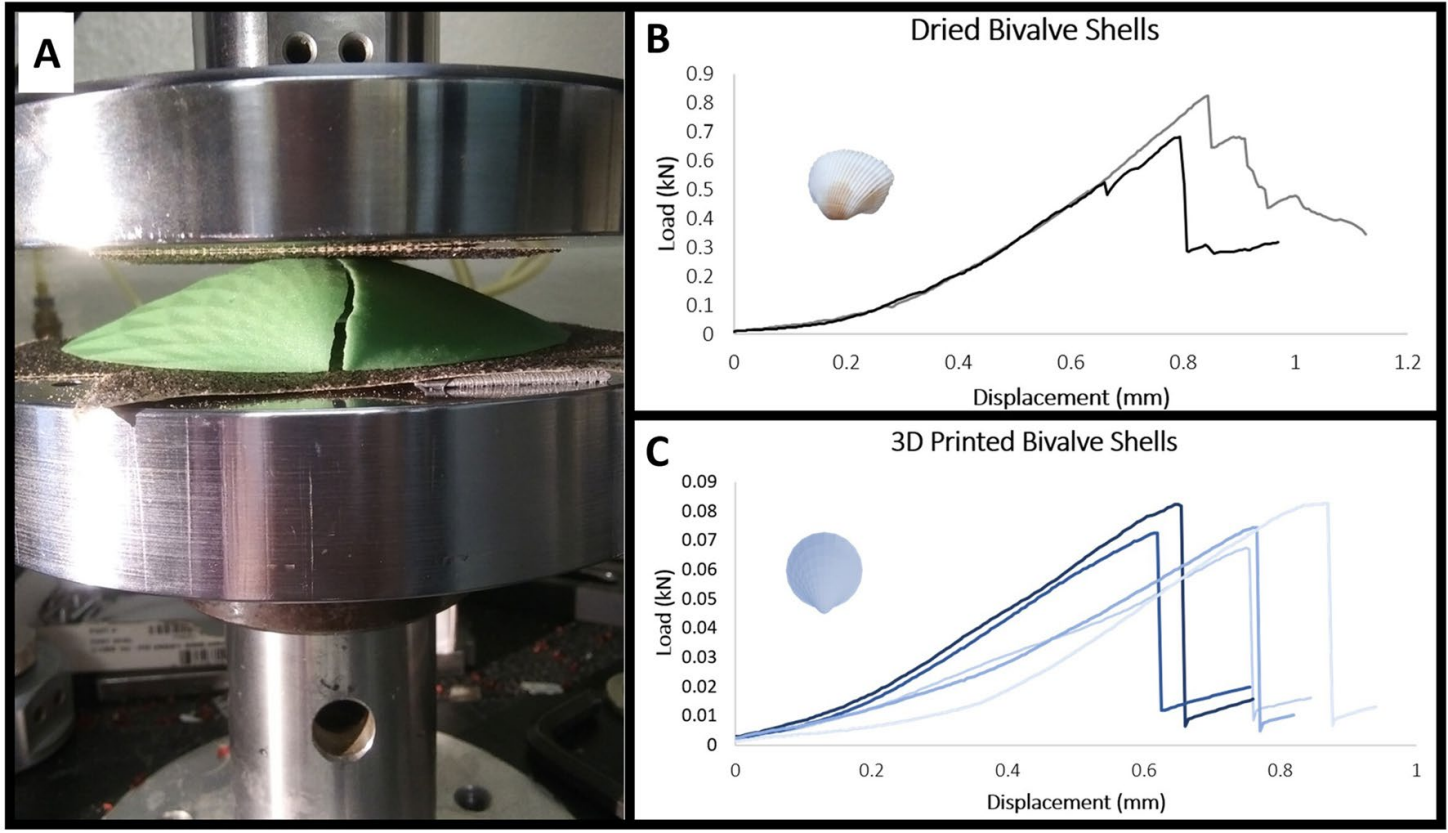
(**A**) Experimental compression of 3D printed bivalve shell model. (**B**) Dried bivalve shells: load (kN) versus displacement (mm) plot. (**C**) 3D printed bivalve shell models: load (kN) versus displacement (mm) plot. Note that both B and C show brittle behavior in compression. This is demonstrated by a small area of plastic behavior followed largely by linear elastic behavior and a subsequent small region of plastic behavior before peak load is reached.

Bivalve shell geometry has been quantified by many different metrics^7,8,24,25^. Some of these metrics include linear measurements of shells (e.g., width of valve)^8^ and mathematical surface models that generate shells by systematically varying parameters of shape like the generating curve and whorl expansion rate^26–28^ (see Table 1). By changing these model parameters shell shapes can be created to represent modern, extinct, and theoretical morphologies (Fig. 1). The ability to create both theoretical and realized morphologies provides the opportunity to study potential functional benefits of different shell geometries. Here, mathematically generated shell shapes were used to experimentally study the adaptive value of different shapes in an idealized system where resistance to compression by a vertebrate predator was treated as the dominant selective pressure. The goal of this study was to isolate and test how (1) shell elongation, (2) whorl expansion rate, and (3) inflation influence the strength of bivalve shells. (1) Shell elongation describes the shape of the ellipse of the shell commissure, the margin upon which new shell material is added during growth. (2) Whorl expansion rate describes how quickly material is added to this margin relative to the coiling of the shell. (3) Inflation refers to the width of a single valve. These parameters, and how they influence bivalve shell shape, are expanded upon below. By testing these parameters, using a theoretical approach to functional morphology, tradeoffs between mechanical and behavioral defenses can be inferred from shapes similar to bivalves of known life modes. Examining the mechanical resistance of different shell shapes allows the evaluation of biological tradeoffs between shapes that more effectively facilitate escape and shapes that may enable less mobility but are more resistant to crushing by a vertebrate predator (Fig. 2). As a result, one may further elucidate the highly restrictive morphological constraints on a very diverse group.

**Table 1:**
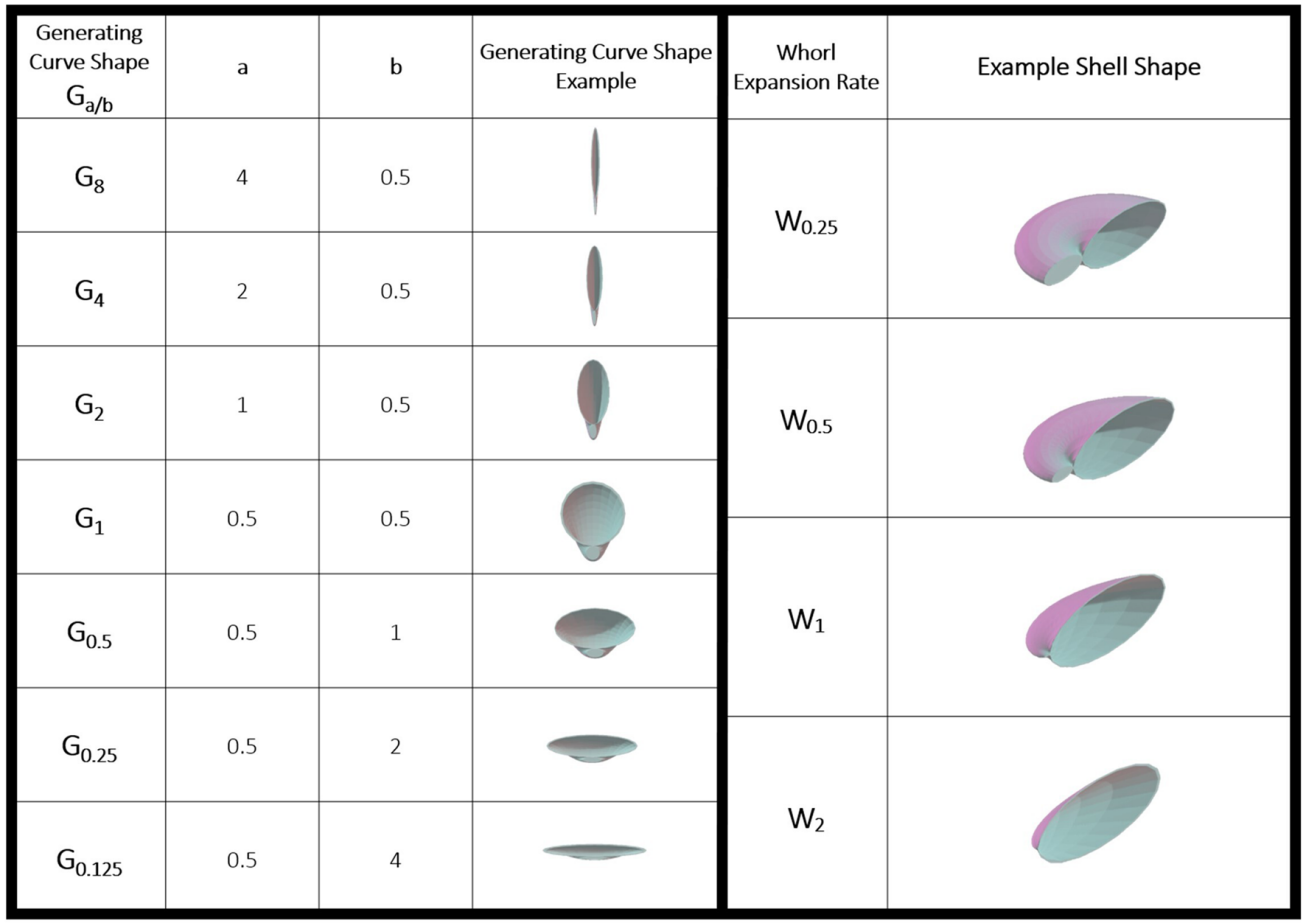
Shell shapes produced by different generating curves using parameters a and b.

Shells for which *a* > *b* are elongated perpendicular to the umbo. Shells for which *a* < *b* are elongated parallel to the umbo (left). Shell shapes produced by different whorl expansion rates (right). Increased whorl expansion rate results in decreased inflation (most inflated shell W_0.25_, least inflated shell W_2_).

## Results

### Shell Elongation

**Figure 3:**
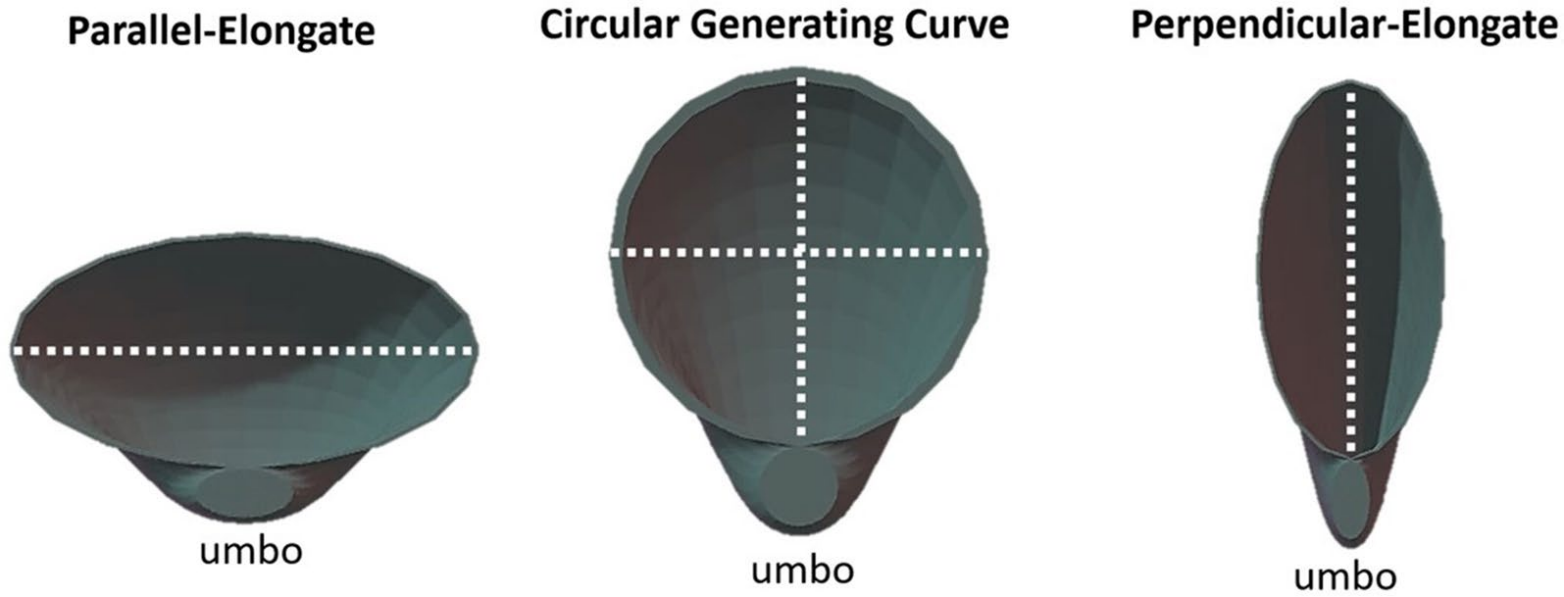
Shell elongation using geometric notation. Parallel-elongate, circular generating curve, and perpendicular-elongate from left to right. All shells oriented with umbo towards the bottom of the figure. White dashed lines denote the longest axis of the generating curve.

**Figure 4:**
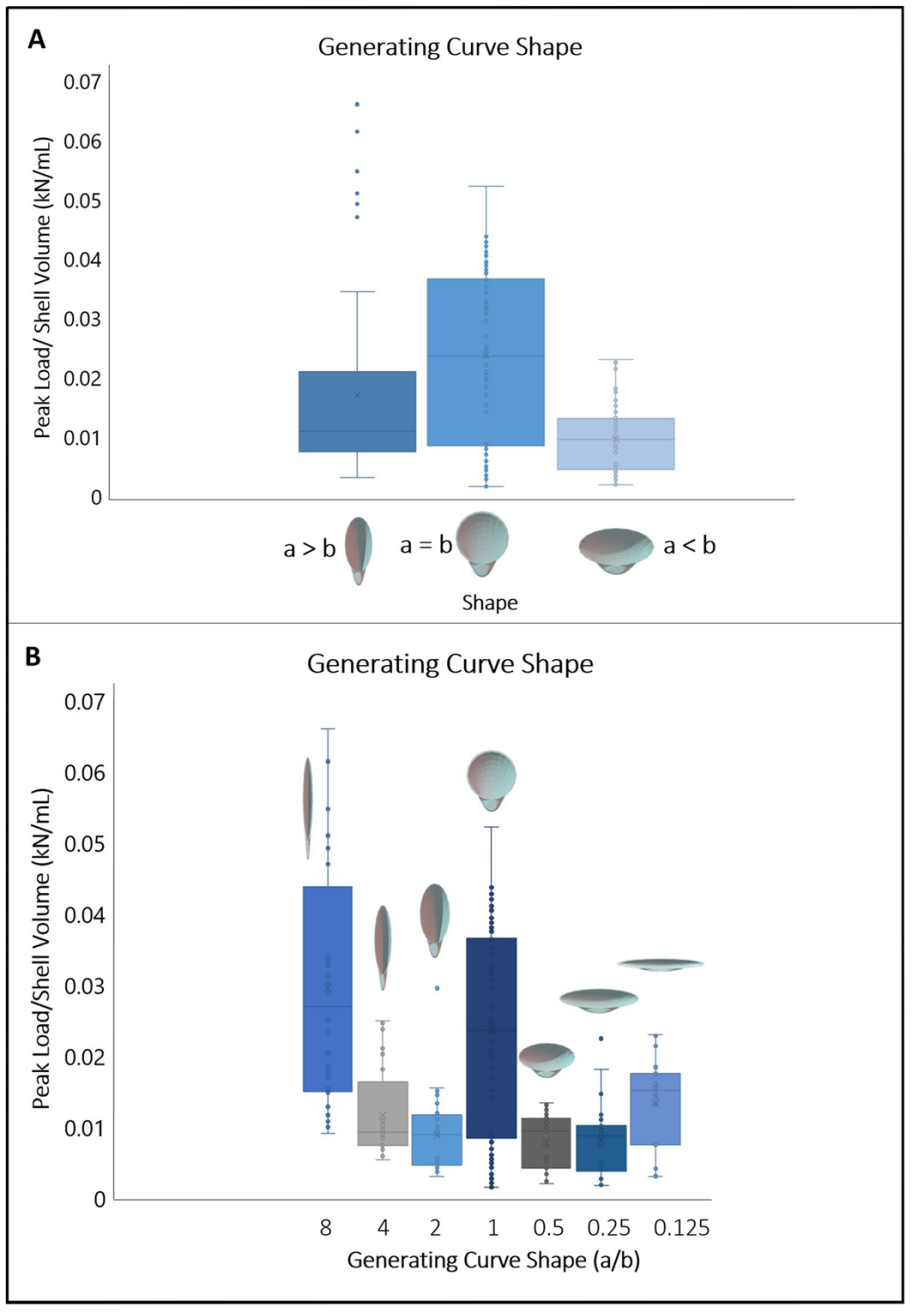
(**A**) Generating curve shape (perpendicular-elongated (a > b) (n = 93), circular (a = b) (n = 88), and parallel-elongated (a < b) (n = 84 )) versus peak load/shell volume (kN/mL) (Shapiro Wilks *p* = 1.102e−13; Kruskal–Wallis *p* = 6.963e−10; see supps. for Dunn Test). (**B**) Generating curve shape (G_8_ (n = 32), G_4_ (n = 32), G_2_ (n = 29), G_1_ (n = 88) G_0.5_ (n = 31) G_0.25_ (n = 30), G_0.125_ (n = 23)) versus peak load/shell volume (kN/mL) (Shapiro Wilks *p* = 8.403e−14; Kruskal–Wallis *p* = 7.129e−16; see supps. for Dunn Test).

The “generating curve” refers to the portion of the shell upon which new material added in the mathematical model. In real shells, this material is secreted by the mantle. Shells were elongated for this study by modifying the generating curves. Generating curve shapes (G_y_ = G_*a/b*_*;* axis perpendicular to the umbo = *a*; axis parallel to the umbo = *b*) were modeled as ellipses to create valves that were elongated perpendicular to the umbo (*a* > *b*, henceforth referred to as perpendicular-elongated), circular (*a* = *b*), or elongated parallel to the umbo (*a* < *b*, henceforth referred to as parallel-elongated) (Fig. 3). The elongation of the generating curve shape in shell models is referred to in these terms, rather than traditional anatomical terms, to highlight shell shape geometrically rather than shell shape orientation based on the adductor muscles. First, shell strength was compared based on direction of elongation (G_*a/b* > 1_, G_1_, G_*a/b* < 1_). The strongest shells, those which sustained the highest peak load per volume of shell material, had circular generating curves (G_1_). The weakest shells, those which sustained the lowest peak load per volume of shell material, were parallel-elongated (G_*a/b* < 1_) (Fig. 4A) (Kruskal–Wallis *p* = 6.963e−10; see S2 for post hoc results). Second, generating curves G_8_, G_4_, G_2_, G_1_, G_0.5_, G_0.25_, and G_0.125_, representing both different directions and magnitudes of elongation, were compared to each other. The strongest shapes, when considered by peak load/volume of shell material, were those that had circular generating curves (G_1_) and the most perpendicular-elongated generating curves (G_8_) (Kruskal–Wallis *p* = 7.129e−16, see Supp. 2 for post hoc results). It should be noted that the extremely perpendicular-elongated shells used here are likely reflective of theoretical morphologies, not appearing in nature due to other constraints such as space for soft tissue. The most parallel-elongated shape (G_0.125_) was also found to be stronger than the other parallel-elongated shells (G_0.5_ and G_0.25_) (Fig. 4B).

### Whorl expansion

**Figure 5:**
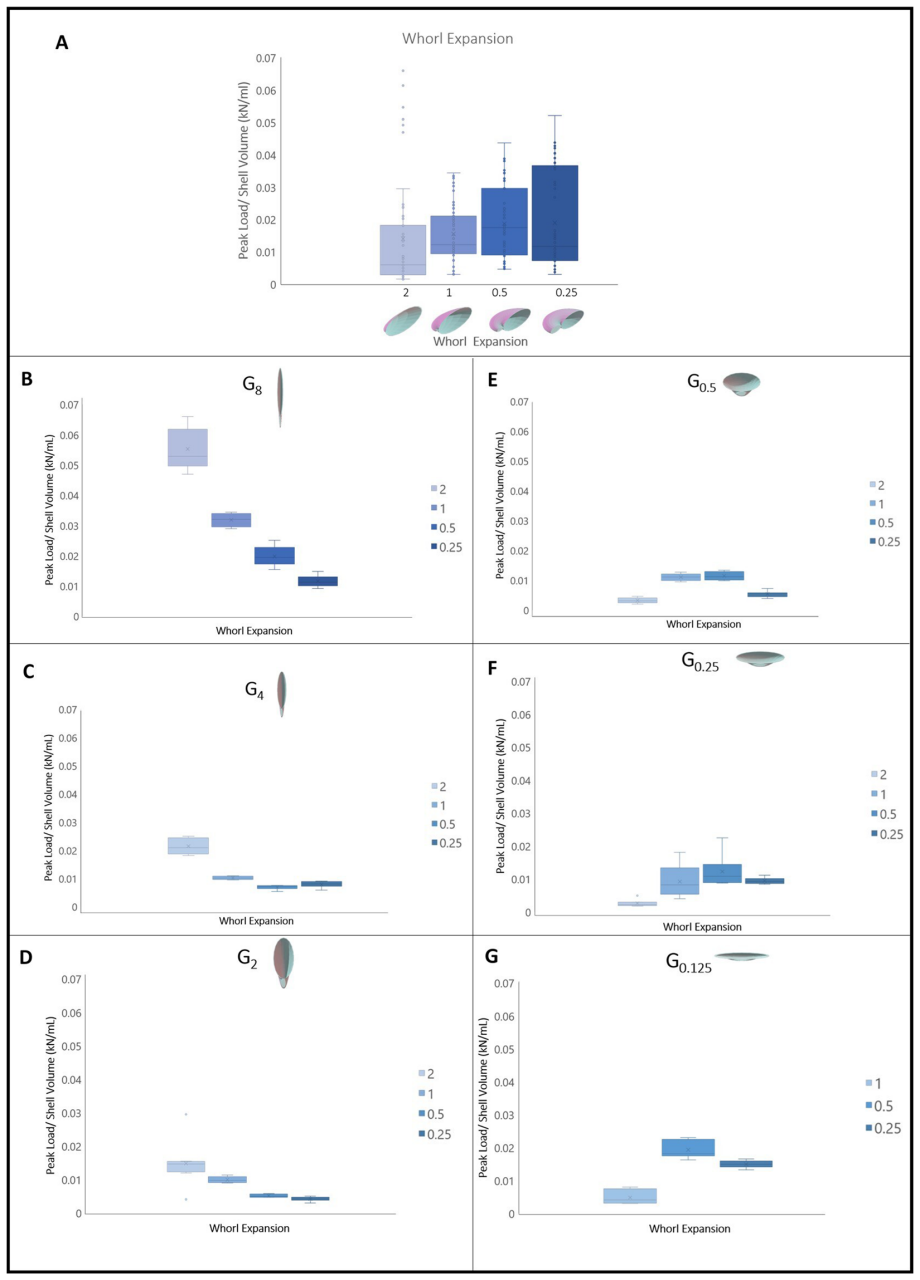
(**A**) Whorl expansion rate (W_0.25_ (n = 71), W_0.5_ (n = 59), W_1_ (n = 71), W_2_ (n = 64)) versus peak load/shell volume (kN/mL) (Shapiro Wilks *p* = 7.621e−14; Kruskal–Wallis *p* = 7.86e−05; see supps. for Dunn Test). (**B**–**G**) Whorl expansion rate versus peak load/shell volume (kN/mL) for various generating curve shapes: (**B**) (G_8_W_0.25_ n = 8), (G_8_W_0.5_ n = 8), G_8_W_1_ n = 8 ), G_8_W_2_ n = 8) (Shapiro Wilks *p* = 0.009822; Kruskal–Wallis *p* = 2.138e−06; see supps. for Dunn Test); (**C**) (G_4_W_0.25_ n = 8), (G_4_W_0.5_ n = 8), G_4_W_1_ n = 8), G_4_W_2_ n = 8) (Shapiro Wilks *p* = 1.041e−05; Kruskal–Wallis *p* = 5.879e−06; see supps. for Dunn Test); (**D**) (G_2_W_0.25_ n = 8), (G_2_W_0.5_ n = 5), G_2_W_1_ n = 8), G_2_W_2_ n = 8) (Shapiro Wilks *p* = 8.54e−05; Kruskal–Wallis *p* = 0.000283; see supps. for Dun Test); (**E**) (G_0.5_W_0.25_ n = 7), (G_0.5_W_0.5_ n = 7), G_0.5_W_1_ n = 7), G_0.5_W_2_ n = 7) (Shapiro Wilks *p* = 0.009189; Kruskal–Wallis *p* = 1.871e−05; see supps. for Dunn Test); (**F**) (G_0.25_W_0.25_ n = 8), (G_0.25_W_0.5_ n = 6), G_0.25_W_1_ n = 8), G_0.25_W_2_ n = 8) (Shapiro Wilks *p* = 0.01294; Kruskal–Wallis *p* = 0.0003822; see supps. for Dunn Test); (**G**) (G_0.125_W_0.25_ n = 8), (G_0.125_W_0.5_ n = 8), G_0.125_W_1_ n = 7) (Shapiro Wilks *p* = 0.01439; Kruskal–Wallis *p* = 6.696e−05; see supps. for Dunn Test)).

Shell strength per volume was not significantly different for W_0.25_ (i.e., whorl expansion rate 0.25), W_0.5_, and W_1_. However, shell strength was lowest in shells with the highest whorl expansion rate (W_2_) (Fig. 5A) (Kruskal–Wallis *p* = 7.86e−05; see S2 for post hoc results). The strengths of shells with the same generating curve shape and differing whorl expansion rates were also compared. Perpendicular-elongate shells, G_8_, G_4_, and G_2,_ (Fig. 5B–D), were stronger with W_2_ than W_0.5_ and W_0.25_ (Kruskal–Wallis *p* = 2.138e−06, *p* = 1.041e−05, *p* = 8.54e−05 respectively, see S2 for post hoc results). Additionally, W_1_ was stronger than W_0.25_ (Fig. 5B–D). In the case of G_4_, W_1_ was also stronger than W_0.5_ (Fig. 5C). For G_0.5_, both W_1_ and W_0.5_ were stronger than W_2_ and W_0.25_ (Fig. 5E) (Kruskal–Wallis *p* = 1.871e−05 see Supp. 2 for post hoc results). When G_0.25_ was tested, W_1_, W_0.5_, and W_0.25_ were all stronger than W_2_ (Fig. 5F) (Kruskal–Wallis *p* = 0.0003822, see Supp. 2 for post hoc results). For shells with G_0.125_, W_0.5_ was stronger than W_0.25_ and W_1._ Additionally, W_0.25_ was stronger than W_1_ (Fig. 5G) (Kruskal–Wallis *p* = 6.696e−05, see S2 for post hoc results).

### Inflation

**Figure 6:**
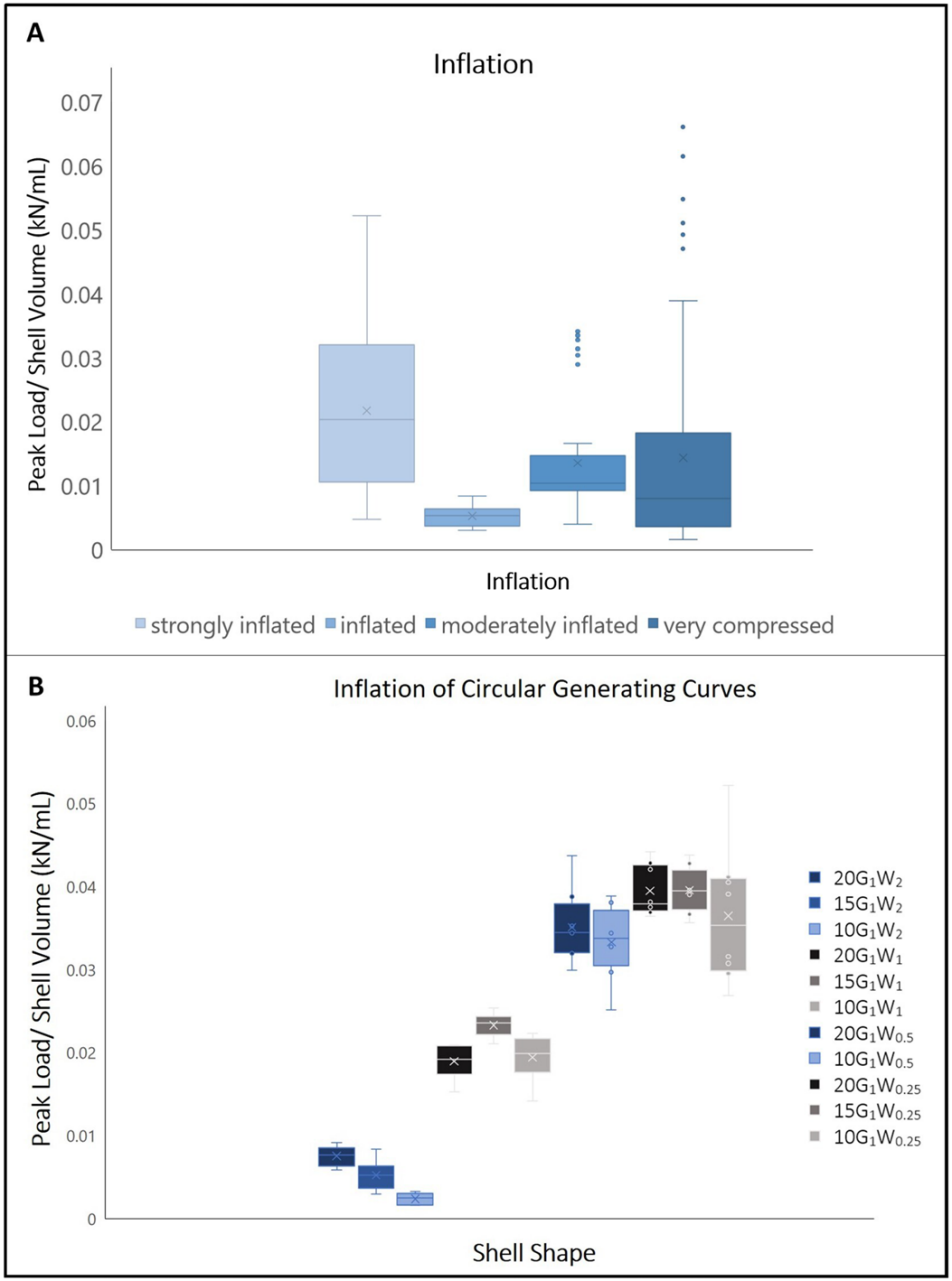
(**A**) Peak load/shell volume (kN/mL) versus inflation (strongly inflated n = 107, inflated n = 9, moderately inflated n = 55, and very compressed n = 95) (Shapiro Wilks *p* = 8.143e−14; Kruskal–Wallis *p* = 2.632e−10; see supps. for Dunn Test). (**B**) Peak load/shell volume (kN/mL) versus inflation of shells with circular generating curves (a = b) (n = 8 for each shape XG_1_W_z_) (Shapiro Wilks *p* = 0.0002167; Kruskal–Wallis *p* = 5.293e−13; see supps. for Dunn Test).

Inflation was calculated following Stanley (1970)^8^ as the ratio of lateral compression (shell height/shell width = maximum dimension perpendicular to shell length/maximum dimension of the two valves perpendicular to the plane of commissure) generating four discrete categories: strongly inflated (≤ 1.29), inflated (1.30–1.49), moderately inflated (1.50–1.69), and very compressed (≥ 2). The strongest shells were those that were strongly inflated. The weakest shells were those that were inflated. There was no significant difference between shells that were moderately inflated and very compressed (Fig. 6A). Shapes with W_0.5_ and W_0.25_ were stronger than shapes with W_2_ and W_1_ when inflated at a single valve width of 10 mm. W_0.5_ and W_0.25_ were stronger than shapes with W_1_ when inflated at a single valve width 20 mm. All W_1_ were stronger than W_2_ at 10 mm. W_1_ was stronger than W_2_ when both were inflated to 15 mm. W_0.25_ at 15 mm and 20 mm was stronger than W_1_ at 15 mm (Fig. 6B).

## Discussion

### Experimental limitations and implications

Many factors are known to contribute to mollusk shell strength including thickness, microstructure, previous shell damage, and ornamentation^19,21,29–31^. Thus, it is challenging to separate the influence of one feature from another when testing the strength of real shells. Fortunately, there are alternative methods by which to model shells^22,32–34^. 3D printing is an effective tool to create model shells that exhibit brittle behavior in compression, serving as an accurate first-order approximation for shell breakage (Fig. 2). 3D printed models are not created to replicate shell microstructure; rather they serve to normalize confounding factors encountered with real shells, like size, variations in thickness, and taphonomy/degradation of microstructure, to isolate variables of interest while maintaining the predominantly brittle behavior seen in real shells. As a result, the failure of different 3D printed shapes under bulk mechanical compression can be used for relative comparisons between morphologies without needing to reproduce the exact magnitude of load to failure of real shells^22,33^ (see “Methods”, Fig. 2). Natural shells ultimately break after cracking through microstructural layers. Analogously, in this study, 3D printed shells cracked predominantly through layers of printed materials, not along printing boundaries, demonstrating an appropriate proxy for shell failure (Figs. 1, 2).

Resistance to compression by a predator can be tested directly using a variety of loading tests, the effectiveness of which has been demonstrated repeatedly by investigators studying topics ranging from predation to climate change^22,33,34^. Compression experiments of bivalve shells, specifically, have been used extensively for this purpose^35–46^. In this study, the mechanical strength of model bivalve shell shapes was analyzed to understand potential defensive value against durophagous (crushing) predators using hypothetical flat teeth and jaws to crush prey (see Crofts and Summers (2014)^22^ for examples of flat crushing morphologies) (Figs. 1, 2). These experiments are most analogous to small shell-crushers with flat dentitions, like fishes (e.g., guitar fishes, stingrays, etc.), rather than large predators like the modern walrus or extinct marine reptiles (e.g., placodonts, mosasaurs) which are so much larger than their prey that the differences in shell strength resulting from shape are likely insignificant. Bivalves are also preyed upon by many other predators including asteroids, gastropods, birds, and mammals^47^. The ability to escape is likely dependent upon predator capabilities, where not all escape mechanisms are equally effective against different predators. The results of these experiments are not intended to be general proxies of predation resistance—they are only applicable as a proxy for predators that use flat crushing dentitions. Additionally, it is important to note that compression of a shell by vertebrate predators is a different mechanical process from compression by an arthropod, as claws localize forces differently than teeth and jaws^48^. Therefore, the experiments used in this study are not meant to model predation by invertebrate durophages. This experimental setup represents an idealized case of shell compression assuming consistent shell thickness and a common predator using a quasistatic loading regime to isolate the influence of shell shape. Therefore, this study makes no conclusions as to the effects of shell shape on strength under conditions of point loading by claws or impact. Furthermore, while bivalve shape is undoubtedly influenced by many factors including fabrication (shell growth and construction)^49^, phylogenetics, location of soft tissue, etc., these experiments were designed to study an idealized case in which defense against vertebrate shell crushing predators is the most important functional constraint on shell shape.

Advantageously, the use of mathematically generated theoretical bivalve shells, rather than real shells, enables testing a range of shapes—some of which can be or have been found in nature—and others that have yet to exist. For example, these methods enable physical testing of morphologies which are only found in the fossil record. Fossils cannot be used for mechanical experimentation due to changes in the integrity of the shell structure resulting from the fossilization process. Furthermore, testing shapes which do not exist due to biological constraints like fabrication, can provide valuable insights when combined with the study of shapes that have evolved naturally. For example, testing theoretical shapes can reveal morphologies that perform better than natural morphologies in specific functional settings. Thus, testing shapes which perform well, but do not exist, is one way to identify the potential influence of evolutionary constraints such as fabrication (shell growth patterns), phylogenetics, or other necessary biological functions. In this study, the extremely perpendicular-elongate shells represent theoretical shapes that are not seen in nature (likely due to space limitations for soft tissue). Developing theoretical physical models also allows for the elimination of many confounding variables that cannot be avoided when testing real shells. The effects of variations in shell thickness and ornamentation, which also play a role in shell strength, can thus be separated from the strength imparted by gross shell shape when theoretical models are generated. However, because overall shell strength is derived from a combination of these many factors (microstructure, thickness, etc.^31^.) the conclusions from these experiments represent a first-order approximation of the effects of shape on shell strength alone. Because the models for this study were generated mathematically, and are therefore not exact replicas of specific taxa, the discussion below aims to address potential tradeoffs based on general shell shapes that could be further studied for specific bivalve taxa in future experiments.

### Shell shape and strength

Three parameters of shell shape were modified for this study. (1) Generating curve shape is both a modeling parameter and biological feature of mollusk shells^27^. In mathematical models, generating curve shape describes the shape that is rotated around an axis to create a surface that represents the overall shell shape. Biologically, the generating curve refers to the portion of the shell upon which new material is secreted by the mantle. In bivalves, this is the shape of the commissure. Thus, by changing the shape of the generating curve in a model bivalve, i.e., changing the shape of the ellipse that generates the surface of the shell, models can be perpendicular-elongated (*a* > *b*) or parallel-elongated (*a* < *b*). In this study, shells with circular generating curves were found to be the strongest, while parallel-elongate shells were the weakest. (2) Whorl expansion rate and shell inflation, both discussed here, can be difficult to disentangle. While the two parameters are related, whorl expansion rate describes how quickly the generating curve of a shell expands, while inflation refers to lateral compression across the valves. Higher whorl expansion rates in bivalve shells lead to greater increases in generating curve size per unit of shell length. This means that bivalves with higher whorl expansion rates are more laterally compressed (less inflated) than bivalves with lower whorl expansion rates. The shells with the highest whorl expansion rate in these experiments, W_2_, were weaker than shells with lower whorl expansion rates. (3) Inflation was measured and categorized following Stanley (1970)^8^, whose shell height/width ratios partitioned the tested models as strongly inflated, inflated, moderately inflated, and very compressed. Strongly inflated shells were stronger than the other levels of inflation tested in these experiments.

Bivalves use burrowing, attaching to substrate, and swimming, among other tactics, as behavioral defenses to evade and avoid predators. It is important to note that bivalves may utilize more than one life mode (e.g., both swimming/hopping and burrowing) and that soft tissue (e.g., muscles) also influences escape abilities^8,23^. However, not all morphologies are conducive to each behavior. Using theoretical tests as a first order approximation for the relative strengths of different shell shapes provides several examples of potential mechanical and behavioral tradeoffs. When scaled by shell material volume, to normalize by the material cost to the organism, the generating curve shapes that produced the strongest shells were circular (Fig. 4A). This shape is frequently seen in pectinids that recline and swim or live attached to substrate^23^. However, while the circular generating curve shape of these shells may increase shell strength, swimming pectinids typically have laterally compressed shells (low inflation, high whorl expansion rate). High whorl expansion rates were observed to decrease shell strength in this study; however, high whorl expansion rates allow for lift and permit swimming to escape predators. The weakness of these shapes indicate that these morphologies have possible tradeoffs. These shell shapes allow for behavioral defenses, but if the organisms are caught, the shapes do not provide very high mechanical resistance to crushing. This is also supported by the observation that swimming pectinids tend to have thin shells which allow for mobility but are not as strong as thick shells^23^. For those which live attached to the substrate, it is possible that the circular generating curve of the shell provides more strength than other shapes might. Some attached bivalves with circular generating curves, like some Spondylidae (which also have defensive spines), have more inflated shells, likely increasing mechanical resistance for bivalves which are unable to escape predators.

Attachment to substrate is also commonly associated with taxa that have shells elongated perpendicular to the umbo (*Mytilus edulis*, *Brachidontes recurvus*, *Brachidontes exustus*). The elongation of the generating curve of these shells results in an increased ventral margin by which to attach. Shells elongated perpendicular to the umbo are not suited for swimming or burrowing^8^. In these experiments, such shells were weaker than those with circular generating curves but stronger than shells with generating curves elongated parallel to the umbo. When considering the contribution of shell shape to shell strength exclusively, perpendicular-elongated shells may demonstrate a tradeoff. Elongation in this direction does not permit escape responses but may provide increased strength to organisms that use their elongated shells to live attached. The compression experiments also indicated that in perpendicular-elongate shells, low whorl expansion rates (resulting in increased inflation) decreased shell strength. This is surprising, as many attached taxa with elongated shells are relatively inflated (*Mytilus edulis*, *Brachidontes recurvus*, *Brachidontes exustus*). In life, the strength of perpendicular-elongate attached shells is likely also due to other factors, like microstructure and increased thickness.

The clearest potential mechanical tradeoff observed was in shells with parallel-elongation which permits burrowing behaviors as a mechanism of escaping predators. Many burrowing bivalves are elongated parallel to the umbo (e.g., *Ensis*, *Mya*) although exceptions exist (e.g. *Dosinia* is a rapid burrower with a very circular generating curve). The direction of shell elongation in these taxa reduces the area of the shell that creates resistance to burrowing, thereby reducing friction and saving energy^8^. The rate at which different taxa burrow is also often related to the inflation of their shells. For example, slow or shallow burrowers tend to have more inflated shells (low whorl expansion rate) that experience increased resistance from sediment during burrowing^8^. Such shallow burrowers also commonly have thicker shells^23^. Prominent exceptions to this are the inflated cardiids, which can burrow rapidly and even jump to evade predators^23^. In contrast, taxa that burrow rapidly tend to have more laterally compressed shells (low inflation, high whorl expansion rate) (e.g., *Ensis*, *Yoldia perprotracta*, *Yoldia limatula*). The streamlined shapes of these compressed shells allow for faster, deeper burrowing to evade predators. When burrowing bivalves become uncovered from the sediment, the ability to burrow again quickly is an important behavioral defense^23^. Since fast burrowing is often a trait of bivalves with compressed shells, organisms that burrow and hide rapidly can afford to have mechanically weaker shells. Yet, those which cannot burrow quickly, and tend to have more inflated shells, likely rely on the increased mechanical strength of their shells for protection if other behavioral defenses (e.g., jumping) are not feasible.

### Future directions

This theoretical study of shell shape in compression demonstrated several potential tradeoffs between mechanical and behavioral defenses against vertebrate shell-crushing predators. However, bivalve shell shapes also show morphological variation outside the scope of this study. In the future, testing new parameters will elucidate further connections between shape and strength. The single model valves used in this study were bilaterally symmetric (symmetric across an axis drawn perpendicular to the umbo of a single valve); however, many valves are not completely symmetric in nature. For example, the perpendicular-elongated shells of attached taxa, like *Mytilus,* are inflated such that the highest point of the shell is not central to the valve. Rather the most inflated parts of these shells are to the anterior or posterior of the umbo. It is likely that the location of the highest point of the valve contributes to shell strength by changing the shape of the curvature of the shell. Related to this observation, the most inflated part of the valve is often located closer to the umbo in epifaunal groups (e.g., *Mytilus edulis*) and further from the umbo in burrowing groups (e.g. *Modiolus modiolus*)^8,23^. Future studies could consider models that modify the placement of the most inflated portion of the valve, as this is where contact is made during compression tests. The umbonal angle of the shell, which is known to relate to swimming capabilities, could also be tested^8^. Tests like those conducted here, which compress one valve, are best applied to bivalves with two valves that are symmetrical to each other (equivalve). Therefore, to understand the strength of those taxa that do not exhibit the equivalve condition it would be necessary to evaluate which valve is the weakest, as the predator may be more likely to break the weaker valve in compression. Based on the results of this study, the weaker valve is likely the more compressed valve.

Here, experimental compression tests of 3D printed shell models were used to understand the extent to which different bivalve shapes provide structural protection against predators. Understanding the influence of shape on shell strength is highly complex because a multitude of shape parameters contribute to shell strength. In this study, the influence of shell elongation, whorl expansion, and inflation were examined. The results suggest that shells with circular generating curves are generally more resistant to crushing than elongated shells. Shells with circular generating curves can be seen in taxa with compressed shells (low inflation) that swim and others that are less compressed but still live epifaunally (though this shape is not exclusive to epifaunal taxa). Elongation, either perpendicular or parallel to the umbo, is a feature often (though, again, not exclusively) seen in groups that use attachment and burrowing, respectively. It would be useful for future studies to quantify the diversity of bivalve generating curve shapes in both modern bivalves and the fossil record to better understand the evolutionary significance of shell elongation. When shells were elongated to any extent, they were less resistant to crushing. These experiments demonstrated potential advantages to extreme elongation, although some of these extreme forms are likely theoretical. Overall, whorl expansion rate did not significantly change shell strength except to cause weakness when whorl expansion rate was very high. Very high whorl expansion rates are often seen in taxa with locomotive abilities such as burrowing and swimming to escape predators. This suggests a tradeoff as the ability to escape comes at the cost of relatively weak armor should the organism be caught. The results also demonstrate that relationships between whorl expansion rate and strength were different depending on the direction of shell elongation (perpendicular or parallel). Thus, different whorl expansion rates were beneficial to shell strength contingent upon whether the organism had parallel-elongation (likely burrowing) or perpendicular-elongation (likely attached). Strongly inflated shells were stronger than their less inflated counterparts; however, some shells that were very compressed were still very strong. This study provides empirical evidence for potential tradeoffs in defensive capabilities of different shell shapes; namely, that the ability to escape or evade predators may result in mechanically weaker shells. However, the results presented here demonstrate that these relationships are not straight-forward. In fact, many different parameters of shell shape can alter the strength of a bivalve shell when acting in concert. Studies in which models are more taxon-specific will be of great importance to elucidate these possible tradeoffs.

## Materials and methods

### Model creation

Single bivalve shell models of 34 different shapes were generated using equations modified from Phillips (2004)^28^ (Fig. S1). These equations describe a surface growing in a logarithmic spiral (similar to models used by Raup (1966))^27^.

$${\text{x}} = {\text{e}}^{{{\text{w}}\cdot{\text{v}}}} \left( {{\text{h}} + {\text{a}}\cdot{\text{cos}}\left( {\text{u}} \right)} \right)\cdot{\text{cos}}\left( {\text{v}} \right)$$

$${\text{y}} = - {\text{e}}^{{{\text{w}}\cdot{\text{v}}}} \left( {{\text{h}} + {\text{a}}\cdot{\text{cos}}\left( {\text{u}} \right)} \right)\cdot{\text{sin}}\left( {\text{v}} \right)$$

$${\text{z}} = {\text{e}}^{{{\text{w}}\cdot{\text{v}}}} \left( {{\text{b}}\cdot{\text{sin}}\left( {\text{u}} \right)} \right)$$

Constants *a* and *b* were modified to change the shape of the generating curve^27^ where *a* describes the axis perpendicular to the umbo and *b* describes the axis parallel to the umbo of the generating curve (see Table 1). *u* parametrizes a complete ellipse for the generating curve while *v* determines the number of coils around the axis. *w* was varied to change whorl expansion rate and *h* was modified to ensure that there was no whorl overlap (see Table 1 and Table S1). These equations were used to make surfaces in Blender 2.8. The subsequently generated surfaces were thickened to 1 mm to create solid objects. In cases in which a circular generating curve was used (*a* = *b*) shell inflation was additionally modified by altering the single valve width (setting valve width to specific measurements e.g., 10 mm). The models were exported as stl files and printed by the company Bluedge using gypsum powder through Colorjet 3D printing (CJP); binder was used to add layers of powder; low viscosity Cyanoacrylate was used to strengthen the prints (see^22^ for similar printing methods) (Fig. 1). Shell types were named as follows XG_y_W_z_ where X represents modified single valve width (for shells with circular generating curves only), *y* represents *a*/*b* and *z* represents whorl expansion rate (*w*).

### Compression experiments

Single 3D printed valves were subjected to compression tests using an Instron Universal Testing Machine (Fig. 2). A 2kN load cell was used to create a quasistatic loading regime using 2 flat plates to mimic the flat crushing teeth or jaws of a vertebrate predator. The top plate was displaced at 3 mm/min and the load was applied to the highest point of the valve mimicking a predator loading across the valves of a shell. The plates were lined with sandpaper to prevent sliding during the experiment. As the top plate was lowered the equal and opposite force of the valve pushing back on the plate was recorded. Single valves were used to ensure stability of the specimen in testing assuming that in an idealized case where two valves could be used the same results would be mirrored by the opposing valve. Load (kN) and displacement (mm) were recorded. The load–displacement curve of brittle materials, like gypsum powder printed shells or real shells, exhibits the following features: some minor plastic behavior at initial strain, followed by a largely linearly elastic behavior as seen by a linear load–displacement curve, another small zone of plastic behavior at large strain that is often a precursor to failure, and finally a peak followed by a drastic drop in load as the object comes out of contact with the plate upon failure. There may also be small dips in the curve demonstrating microcracks (Fig. 2). While the magnitude of load at failure differs between real shells and 3D printed shells, and even between real shells, brittle behavior dominates in all cases of compression as seen in load versus displacement curves^22,33,50,51^.

### Statistical analyses

Peak load (kN) was measured as the highest load sustained before failure, which was signified by a rapid drop in load as the plate lost contact with the specimen. For all analyses, peak loads were divided by the amount of material used to make each shell shape (mL) to normalize load sustained by the material cost to the theoretical organism (i.e., dividing by the shell material allowed comparisons based on the amount of shell material an organism would need to invest to create the shape). For each analysis, R was used to conduct a Shapiro–Wilks normality test (*p* < 0.1), followed by Kruskal–Wallis rank sum tests (α = 0.05) (none of the samples were normally distributed) and finally a post-hoc Dunn’s Test (α = 0.05) (n = 5 to 8; see Figs. 5, 6A and S2 for details).

## Supplementary information

Supplementary Information.
