# Supplementary material for: Experimental tests of bivalve shell shape reveal potential tradeoffs between mechanical and behavioral defenses
Source: Sci Rep. 2020 Nov 10;10:19425. doi: 10.1038/s41598-020-76358-x (PMC7655838; doi:10.1038/s41598-020-76358-x)
Supplement: Supplementary file 1 — Supplementary Information. [file 41598_2020_76358_MOESM1_ESM.pdf]

Figure S1

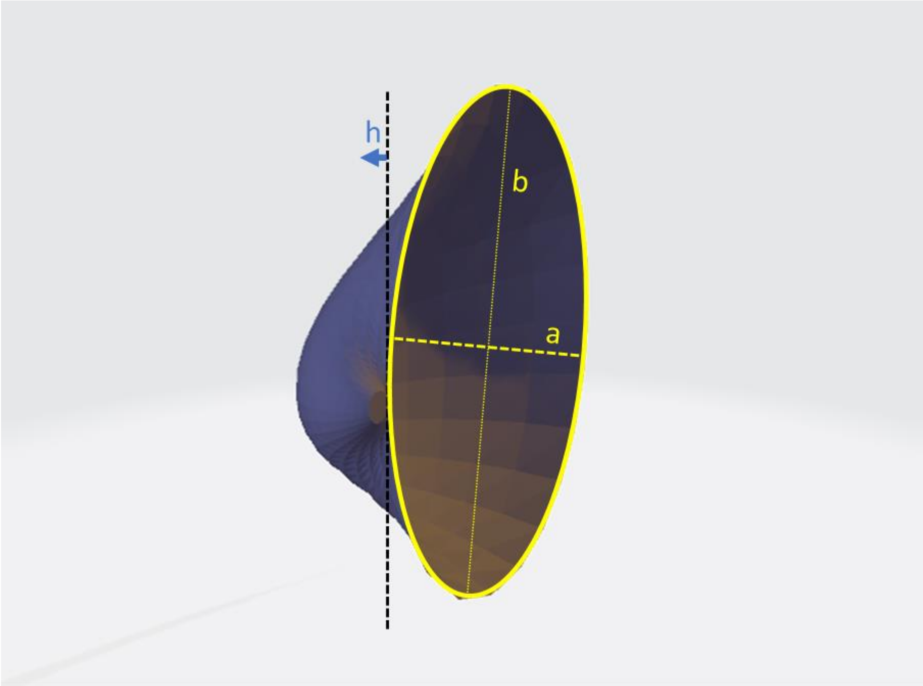

Figure S1: Image showing model generation. Surface is constructed by sweeping a generating curve (yellow oval) around a coiling axis (black dotted line). Shape of the generating curve is determined by axes *a* and *b*. Parameter *h* creates distance (blue arrow) between the generating curve and the coiling axis (if h = 0 then the generating curve touches the coiling axis).

Table S1

Shape Name	a/b	h	a	b	w	set height (mm)
$G_8W_{0.25}$	8	4	4	0.5	0.25	
$G_8W_{0.5}$	8	4	4	0.5	0.5	
G_8W_1	8	4	4	0.5	1	
G_8W_2	8	4	4	0.5	2	
$G_4W_{0.25}$	4	2	2	0.5	0.25	
$G_4W_{0.5}$	4	2	2	0.5	0.5	
G_4W_1	4	2	2	0.5	1	
G_4W_2	4	2	2	0.5	2	
$G_2W_{0.25}$	2	1	1	0.5	0.25	
$G_2W_{0.5}$	2	1	1	0.5	0.5	
G_2W_1	2	1	1	0.5	1	
G_2W_2	2	1	1	0.5	2	
$10G_1W_{0.25}$	1	0.5	0.5	0.5	0.25	10
$15G_1W_{0.25}$	1	0.5	0.5	0.5	0.25	15
$20G_1W_{0.25}$	1	0.5	0.5	0.5	0.25	20
$10G_1W_{0.5}$	1	0.5	0.5	0.5	0.5	10
$20G_1W_{0.5}$	1	0.5	0.5	0.5	0.5	20
$10G_1W_1$	1	0.5	0.5	0.5	1	10
$15G_1W_1$	1	0.5	0.5	0.5	1	15
$20G_1W_1$	1	0.5	0.5	0.5	1	20
$10G_1W_2$	1	0.5	0.5	0.5	2	10
$15G_1W_2$	1	0.5	0.5	0.5	2	15
$20G_1W_2$	1	0.5	0.5	0.5	2	20
$G_{0.5}W_{0.25}$	0.5	0.5	0.5	1	0.25	
$G_{0.5}W_{0.5}$	0.5	0.5	0.5	1	0.5	
$G_{0.5}W_1$	0.5	0.5	0.5	1	1	
$G_{0.5}W_2$	0.5	0.5	0.5	1	2	
$G_{0.25}W_{0.25}$	0.25	0.5	0.5	2	0.25	
$G_{0.25}W_{0.5}$	0.25	0.5	0.5	2	0.5	
$G_{0.25}W_1$	0.25	0.5	0.5	2	1	
$G_{0.25}W_2$	0.25	0.5	0.5	2	2	
$G_{0.125}W_{0.25}$	0.125	0.5	0.5	4	0.25	
$G_{0.125}W_{0.5}$	0.125	0.5	0.5	4	0.5	
$G_{0.125}W_1$	0.125	0.5	0.5	4	1	

Table S1: Bivalve model inputs: shape name, elongation (a/b), h, a, b, w, and height if modified.

S2 Significant Statistical Results

Figure 3A

Shapiro Wilks: W = 0.87802, p-value = 1.102e-13; *Kruskal-Wallis rank sum test:* Kruskal-Wallis chi-squared = 42.17, df = 2, p-value = 6.963e-10

Dunn's Multiple Comparison Test

List of pairwise comparisons: significant p-values

(a = b) – (a > b) : (0.0008)

(a = b) – (a < b) : (0.0000)

(a > b) – (a < b) : (0.0003)

Figure 3B

Shapiro Wilks: W = 0.87611, p-value = 8.403e-14; *Kruskal-Wallis rank sum test:* Kruskal-Wallis chi-squared = 83.385, df = 6, p-value = 7.129e-16

Dunn's Multiple Comparison Test

List of pairwise comparisons: significant p-values

G_2 – G_8 : (0.0000)

G_4 - G_8 : (0.0000)

G_2 – G_1 : - (0.0000)

G_4 – G_1: (0.0002)

G_8 - $G_{0.5}$: (0.0000)

G_1 - $G_{0.5}$: (0.0000)

G_8 - $G_{0.25}$: (0.0000)

G_1 - $G_{0.25}$: (0.0000)

G_8 - $G_{0.125}$: (0.0005)

G_1 - $G_{0.125}$: (0.0098)

$G_{0.5}$ - $G_{0.125}$: (0.0151)

$G_{0.25}$ - $G_{0.125}$: (0.0119)

Figure 4A

Shapiro Wilks: W = 0.87542, p-value = 7.621e-14; *Kruskal-Wallis rank sum test:* Kruskal-Wallis chi-squared = 21.611, df = 3, p-value = 7.86e-05

Dunn's Multiple Comparison Test

List of pairwise comparisons: significant p-values

$W_{0.25}$ - W_2 : (0.0002)

$W_{0.5}$ - W_2 : (0.0000)

W_1 - W_2 : (0.0003)

Figure 4B

Shapiro-Wilk normality test: W = 0.90534, p-value = 0.009822; *Kruskal-Wallis rank sum test:* Kruskal-Wallis chi-squared = 29.096, df = 3, p-value = 2.138e-06

Dunn's Multiple Comparison Test

List of pairwise comparisons: significant p-values

$G_8W_{0.25}$ - G_8W_1 : (0.0003)

$G_8W_{0.25}$ - G_8W_2 : (0.0000)

$G_8W_{0.5}$ - G_8W_2 : (0.0003)

Figure 4C

Shapiro-Wilk normality test: W = 0.76055, p-value = 1.041e-05; *Kruskal-Wallis rank sum test:* Kruskal-Wallis chi-squared = 27.003, df = 3, p-value = 5.879e-06

Dunn's Multiple Comparison Test

List of pairwise comparisons: significant p-values

$G_4W_{0.25}$ - G_4W_1 : (0.0176)

$G_4W_{0.5}$ - G_4W_{01} : (0.0013)

$G_4W_{0.25}$ - G_4W_2 : (0.0001)

$G_4W_{0.5}$ - G_4W_2 : (0.0000)

Figure 4D

Shapiro-Wilk normality test: W = 0.79446, p-value = 8.54e-05; *Kruskal-Wallis rank sum test:* Kruskal-Wallis chi-squared = 18.928, df = 3, p-value = 0.000283

Dunn's Multiple Comparison Test

List of pairwise comparisons: significant p-values

$G_2W_{0.25}$ - G_2W_1 : (0.0015)

$G_2W_{0.25}$ - G_2W_2 : (0.0000)

$G_2W_{0.5}$ - G_2W_2 : (0.0062)

Figure 4E

Shapiro-Wilk normality test: W = 0.90159, p-value = 0.009189; *Kruskal-Wallis rank sum test:* Kruskal-Wallis chi-squared = 24.601, df = 3, p-value = 1.871e-05

Dunn's Multiple Comparison Test

List of pairwise comparisons: significant p-values

$G_{0.5}W_{0.25}$ - $G_{0.5}W_{0.5}$: (0.0038)

$G_{0.5}W_{0.25}$ - $G_{0.5}W_1$: (0.0081)

$G_{0.5}W_{0.5}$ - $G_{0.5}W_2$: (0.0000)

$G_{0.5}W_1$ - $G_{0.5}W_2$: (0.0000)

Figure 4F

<u>Shapiro-Wilk normality test:</u> W = 0.90501, p-value = 0.01294; *<u>Kruskal-Wallis rank sum test:</u>* Kruskal-Wallis chi-squared = 18.296, df = 3, p-value = 0.0003822

<u>Dunn's Multiple Comparison Test</u>

List of pairwise comparisons: significant p-values

$G_{0.25}W_{0.25}$ - $G_{0.25}W_{2}$: (0.0005)

$G_{0.25}W_{0.5}$ - $G_{0.25}W_{2}$: (0.0000)

$G_{0.25}W_{1}$ - $G_{0.25}W_{2}$: (0.0023)

Figure 4G

<u>Shapiro-Wilk normality test:</u> W = 0.88396, p-value = 0.01439; *<u>Kruskal-Wallis rank sum test:</u>* Kruskal-Wallis chi-squared = 19.223, df = 2, p-value = 6.696e-05

<u>Dunn's Multiple Comparison Test</u>

List of pairwise comparisons: significant p-values

$G_{0.125}W_{0.25}$ - $G_{0.125}W_{0.5}$: (0.0111)

$G_{0.125}W_{0.25}$ - $G_{0.125}W_{1}$: (0.0149)

$G_{0.125}W_{0.5}$ - $G_{0.125}W_{1}$: (0.0000)

Figure 5A

<u>Shapiro-Wilk normality test:</u> W = 0.87589, p-value = 8.143e-14; *<u>Kruskal-Wallis rank sum test:</u>* Kruskal-Wallis chi-squared = 47.568, df = 3, p-value = 2.632e-10

<u>Dunn's Multiple Comparison Test</u>

List of pairwise comparisons: significant p-values

----

inflated - moderately inflated : (0.0035)

inflated - strongly inflated : (0.0000)

moderately inflated - strongly inflated : (0.0004)

inflated - very compressed : (0.0242)

strongly inflated - very compressed : (0.0000)

Figure 5B

Shapiro-Wilk normality test: W = 0.93276, p-value = 0.0002167; *Kruskal-Wallis rank sum test:* Kruskal-Wallis chi-squared = 79.883, df = 10, p-value = 5.293e-13

Dunn's Multiple Comparison Test

List of pairwise comparisons: significant p-values

$10G_1W_{0.25}$ - $10G_1W_1$: (0.0046)

$15G_1W_{0.25}$ - $10G_1W_1$: (0.0004)

$20G_1W_{0.25}$ - $10G_1W_1$: (0.0006)

$10G_1W_{0.5}$ - $10G_1W_1$: (0.0173)

$20G_1W_{0.5}$ - $10G_1W_1$: (0.0089)

$15G_1W_{0.25}$ - $15G_1W_1$: (0.0060)

$20G_1W_{0.25}$ - $15G_1W_1$: (0.0083)

$10G_1W_{0.25}$ - $20G_1W_1$: (0.0031)

$15G_1W_{0.25}$ - $20G_1W_1$: (0.0003)

$20G_1W_{0.25}$ - $20G_1W_1$: (0.0004)

$10G_1W_{0.5}$ - $20G_1W_1$: (0.0122)

$20G_1W_{0.5}$ - $20G_1W_1$: (0.0061)

$10G_1W_{0.25}$ - $10G_1W_2$: (0.0000)

$15G_1W_{0.25}$ - $10G_1W_2$: (0.0000)

$20G_1W_{0.25}$ - $10G_1W_2$: (0.0000)

$10G_1W_{0.5}$ - $10G_1W_2$: (0.0000)

$20G_1W_{0.5}$ - $10G_1W_2$: (0.0000)

$10G_1W_1$ - $10G_1W_2$: (0.0119)

$15G_1W_1$- $10G_1W_2$: (0.0010)

$20G_1W_1$ - $10G_1W_2$: (0.0169)

$10G_1W_{0.25}$ - $15G_1W_2$: (0.0000)

$15G_1W_{0.25}$ - $15G_1W_2$: (0.0000)

$20G_1W_{0.25}$ - $15G_1W_2$: (0.0000)

$10G_1W_{0.5}$ - $15G_1W_2$: (0.0001)

$20G_1W_{0.5}$ - $15G_1W_2$: (0.0000)

$15G_1W_1$- $15G_1W_2$: (0.0080)

$10G_1W_{0.25}$ - $20G_1W_2$: (0.0001)

$15G_1W_{0.25}$ - $20G_1W_2$: (0.0000)

$20G_1W_{0.25}$ - $20G_1W_2$: (0.0000)

$10G_1W_{0.5}$ - $20G_1W_2$: (0.0006)

0.5 at 20 - $20G_1W_2$: (0.0002)
